# Reduced COVID-19 morbidity and mortality in hemodialysis patients across the various Omicron sublineages—A retrospective analysis

**DOI:** 10.3389/fpubh.2023.1218188

**Published:** 2023-08-10

**Authors:** Max Schuller, Noemi Elisabeth Ginthör, Astrid Paller, Maximilian Waller, Martin Köstenbauer, Nikolaus Gustav Oskar Schreiber, Corinna Schabhüttl, Kathrin Mischinger, Hildegard Hafner-Giessauf, Alexander R. Rosenkranz, Philipp Eller, Kathrin Eller

**Affiliations:** ^1^Division of Nephrology, Department of Internal Medicine, Medical University of Graz, Graz, Austria; ^2^Department of Medicine I, Klinik Favoriten, Vienna, Austria; ^3^Dialyse Institut Feldbach, Feldbach, Austria; ^4^Clinical Division of Nephrology and Dialysis, Internal Medicine III, Medical University of Vienna, Vienna, Austria; ^5^Department of Internal Medicine, Krankenhaus der Barmherzigen Brüder Graz, Graz, Austria; ^6^Dialysezentrum Graz-West, Graz, Austria; ^7^Dialyseinstitut Gießauf Gmbh, Graz, Austria; ^8^Intensive Care Unit, Department of Internal Medicine, Medical University of Graz, Graz, Austria

**Keywords:** BA.4/5, COVID-19, hemodialysis, Omicron, vaccination

## Abstract

**Introduction:**

Hemodialysis (HD) patients are a COVID-19 high risk population due to comorbidities and impaired immune response. Vaccines, advent of effective treatment and the emergence of novel variants have fundamentally changed the pandemic. We aimed to assess temporal changes of COVID-19 in HD patients of our catchment area, and risk factors for severe and fatal course.

**Methods and materials:**

We retrospectively collected data from 274 patients admitted to the Medical University Graz, Austria for HD between 1st of May 2020 and 31st of August 2022. We analyzed clinical and demographic data between different COVID-19 waves and assessed factors associated with hospitalization, ICU admission and mortality by logistic regression. To further evaluate the dialysis at-risk population, we collected demographic and vaccination data between August 2021 and August 2022.

**Results:**

Time of infection and SARS-CoV-2 sequencing data allowed for distinction of five separate waves of infection with different impact on the dialysis population: While in the initial four waves frequencies of hospitalization, necessity of critical care and mortality were around 60%, 10% and 20%, respectively. These events became rare during the large fifth wave, when Omicron had become the dominant variant. Although only 16.9% had to be hospitalized, this resulted in 29 hospital admissions, due to the high prevalence of COVID-19 during the Omicron era. Furthermore, we observed similar clinical outcomes with BA.4/5 as with BA.1/BA.2 Omicron sublineages. The proportion of previously infected increased simultaneously with the number of vaccination doses in our dialysis population. Vaccination at time of positivity and infection with an Omicron variant conferred protection against hospitalization and mortality in univariate analysis, but only infection with an Omicron variant remained a robust predictor for these outcomes in multivariable analysis.

**Discussion:**

While a fourth of our at-risk population became infected during the Omicron wave, mortality was almost non-existent. Several concomitant factors have contributed to the decrease of COVID-19 severity in HD patients. This trend appears to be continued with BA.4/5, which was equally mild as BA.1 and BA.2 in our well vaccinated dialysis population.

## 1. Introduction

Individuals on hemodialysis (HD) have been at an increased risk of contracting SARS-CoV-2 (1), and of a severe course of COVID-19 (2, 3). While initial reports suggested a mortality rate of almost 30% (4), the pandemic has fundamentally changed since its emergence in late 2019, especially due to the appearance of variants of concern (VoC).

VoC are a consequence of ongoing mutations and constant selection, and are characterized by increased immune escape, rapid transmission and/or more severe disease.

Each novel VoC dealt a different set of cards, challenging health care systems around the world to rapidly adapt to each VoC's characteristics (5). The most “successful” VoC have been B.1.617.2 (Delta) and B.1.1.529 (Omicron) (6), which differ profoundly in transmissibility and virulence. While Delta posed a major threat to the infected, morbidity and mortality have been low with Omicron (7, 8), which has become the dominant variant due to its ability to rapidly spread (9) and partial escape from antibodies (10). While accumulation of mutations may be slowed by preventive measures, genetic transformation of SARS-CoV-2 cannot be halted completely, and concerns remain high, that a more aggressive variant may arise (11).

Natural immunity after COVID-19 offers a certain degree of protection from future infections in HD patients (12), but it potentially comes at a high cost. Therefore, major efforts have been undertaken to expedite the distribution of vaccines. Although the prospect of sterile immunity has diminished considering novel variants, vaccinations are an effective measure against severe and fatal COVID-19 (13). There is now a large body of evidence displaying that SARS-CoV-2 vaccination elicits a dampened, but still measurable serological response in individuals on HD, who were excluded from initial trials (14). However, frequent booster shots may be necessary in this population to combat waning immunity and non-responders (15). VoC with significant immune escape have further added to the problem (10).

Apart from preventive measures, effective treatments, including antivirals and anti-inflammatory agents have been added to the clinician's armamentarium (16).

In this study, we collected data from SARS-CoV-2 positive HD patients from the first recorded SARS-CoV-2 infection in March 2020 until August 2022. Infected patients were referred to our dialysis center in Graz from our catchment area which consists of around 470 HD patients. We aimed to assess temporal changes between pandemic waves and the influence of potential risk and protective factors on outcomes like hospitalization and mortality. Furthermore, we investigated potential differences between the Omicron sublineages BA.1/BA.2 and BA.4/5.

## 2. Materials and methods

### 2.1. Data collection

**Figure 1:**
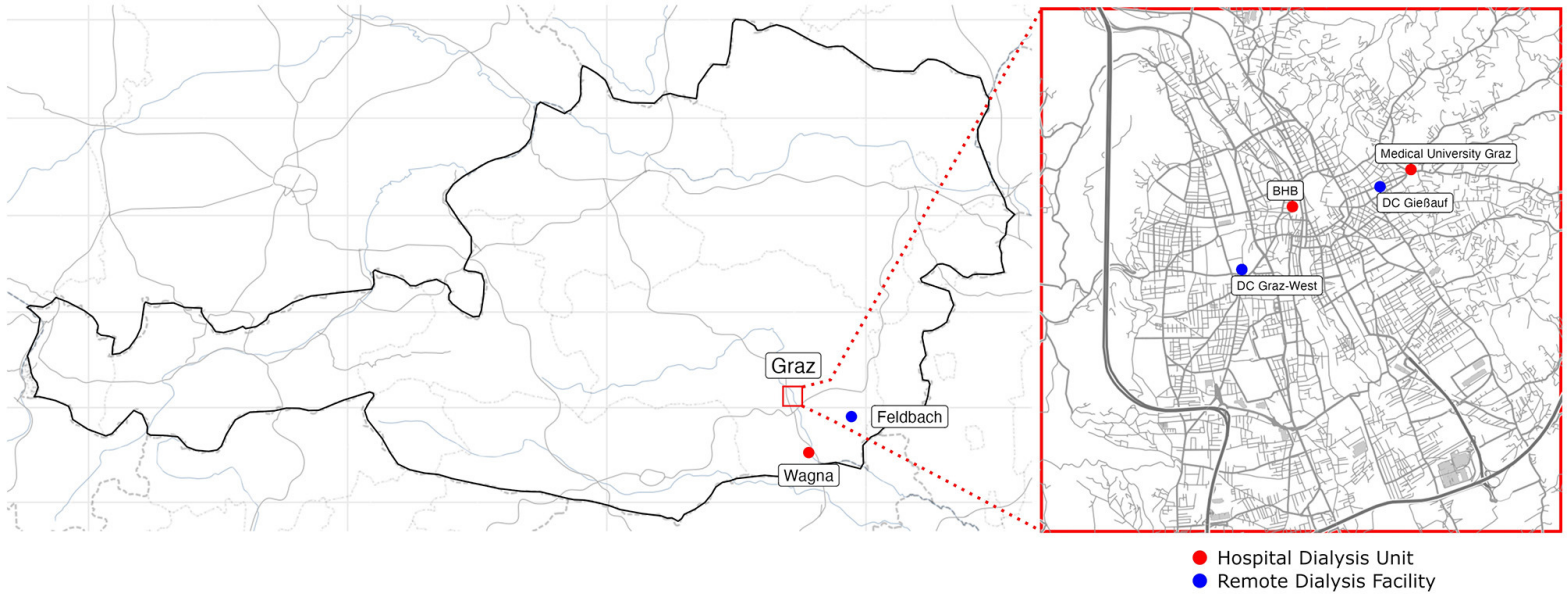
Participating dialysis centers are depicted on a geographical map of Austria with an auxiliary map of Graz (right side). Remote dialysis facilities are shown as blue dots and hospital-based dialysis units are represented as red dots.

HD patients were screened for SARS-CoV-2 positivity by antigen testing before each routine dialysis at the Medical University Graz, a tertiary hospital, two secondary care centers and three remote dialysis facilities (Figure 1). All antigen tests adhered to quality criteria provided by WHO, but different kits were used depending on local availability (17). They were additionally tested whenever SARS-CoV-2 infection was clinically suspected. When tested positively, HD patients were transferred to the Medical University of Graz dialysis unit for disease control reasons and confirmation of infection by PCR testing following a nasal swab. Consequently, patients with (1) PCR confirmed SARS-CoV-2 infection, (2) on hemodialysis prior to infection, and (3) on hemodialysis for at least three months in total, were recruited from March 2020 to 31st of August 2022. Duration of SARS-CoV-2 positivity was defined as the interval between the first positive PCR result and the date of the first polymerase chain reaction (PCR) with a CT (cycle threshold) > 30 followed by a consecutive PCR with increasing CT.

Electronical medical records were reviewed for hospitalization, ICU admission, mortality and treatment. “Hospitalization” was defined as hospitalized while SARS-CoV-2 PCR positive, and those who were already hospitalized at the time of viral contraction were excluded. Any admission to the intensive care unit during hospitalization was recorded as “ICU admission”. We specified “COVID-19 related mortality” as death within 30 days of SARS-CoV-2 positivity.

The following variables were documented as dichotomous events: usage of antibiotics, antivirals or corticosteroids as COVID-19 treatment; Cardiovascular disease was defined as previous coronary artery disease, peripheral artery disease or cerebrovascular disease; Congestive heart failure (regardless of ejection fraction); Diabetes mellitus (any type); Pulmonary disease was defined as interstitial, obstructive or vascular lung disease; Kidney transplantation prior to HD dependency; Immunosuppression comprised the regular intake of calcineurin inhibitors, antimetabolites, prednisolone (or equivalent) above 10 milligrams daily or treatment with immunomodulatory biologicals at the time of infection.

“Waves” could be distinguished by time and by SARS-CoV-2 sequencing results.

Additionally, we collected clinical and vaccination data of the at-risk HD population between 31st of August 2021 and 31st of August 2022, as provided by the individual dialysis units and/or hospitals (Figure 1). Patients who were on dialysis for at least 3 months were included, and data was censored on the 31st of August 2022. Previous SARS-CoV-2 positivity was defined as any SARS-CoV-2 positive PCR test results within the electronical medical records, regardless of dialysis dependency at the time of positivity.

The study was approved by the ethics committee of the Medical University of Graz (EK 34-372ex21/22).

### 2.2. Statistical analysis

Descriptive data are given as median with interquartile range for continuous variables, and absolute numbers and percentages for categorical variables. Clinical characteristics of SARS-CoV-2 infected patients were compared between waves by Kruskal-Wallis test or Chi-Square test, depending on the variable. Weekly prevalence and weekly incidence of SARS-CoV-2 were calculated by dividing the number of currently infected and newly infected individuals, respectively, by the total number of prevalent dialysis patients during the same week.

Univariate logistic regression was used to identify risk factors for dichotomous outcome events like hospitalization, ICU admission and mortality. For multivariable analysis of these outcomes, all variables with a significant impact in univariate analysis were included.

Statistical analysis was performed using SPSS 29 (IBM, Endicott, NY, USA) or RStudio (PBC, Boston, MA, USA).

## 3. Results

### 3.1. Analysis of SARS-CoV-2 positive patient cases over time reveals five distinguishable waves

**Figure 2:**
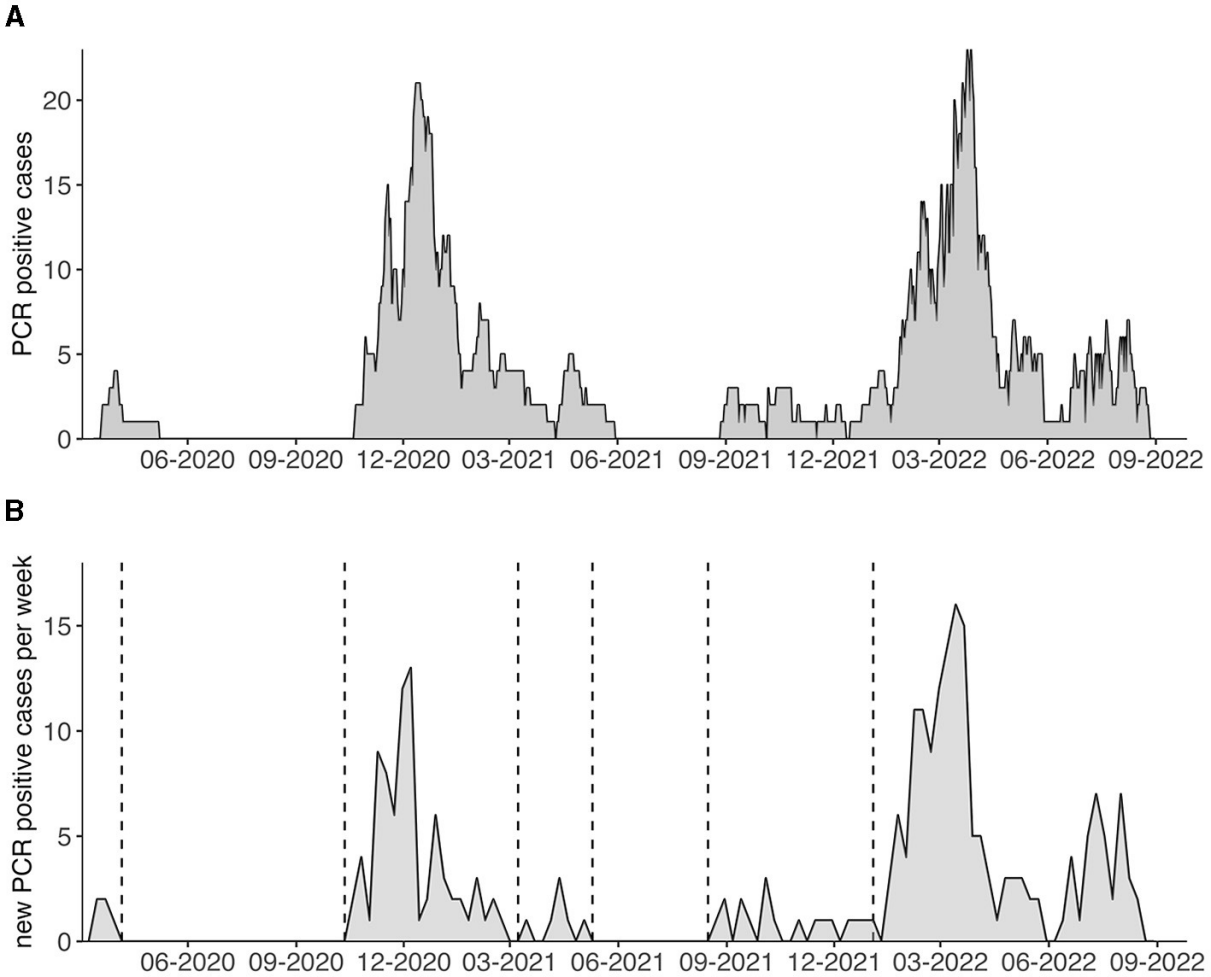
**(A)** PCR confirmed SARS-CoV-2 positive hemodialysis patients in our catchment area are displayed over time and for the duration of PCR positivity. **(B)** Weekly new PCR confirmed SARS-CoV-2 cases are depicted. Vertical dotted lines indicate different waves.

By plotting all 274 SARS-CoV-2 positive patients, who met the inclusion criteria, over time, we could identify five separate “waves” of infections from our first recorded case on 20th of March 2020 until the 31^st^ of August 2022 (Figures 2A, B). This segmentation is supported by the available SARS-CoV-2 genome sequencing data, which shows no overlap of variants between these waves (Table 1). Clinical characteristics, disease outcomes and vaccination status at the time of infection are compared between the five waves in Table 1.

**Table 1:** Clinical characteristics, SARS-CoV-2 sequencing, vaccination status at time of infection, COVID-19 treatment and related outcomes are displayed as absolute and relative frequencies or medians with interquartile range.

**Wave**	**1**	**2**	**3**	**4**	**5**
Time period^a^	20-Mar-2020– 31-Mar-2020	21-Oct-2020– 22-Feb-2021	16-Mar-2021– 05-May-2021	28-Aug-2021– 26-Dec-2021	02-Jan-2022– 31-Aug-2022
N	5	79	7	16	167
Age (years)	71 (45.5–85)	74 (65–79)	74 (70–77)	67.5 (57.8–80.8)	71 (56–78)
Male gender (%)	3 (60)	35 (44.3)	5 (71.4)	8 (50)	97 (58.1)
BMI (kg/m2)^b^	25.6 (22.4–26.9)	25.6 (22.1–30.1)	23 (21.1–26.9)	26 (21.4–30.4)	26.1 (22.7–30.7)
**Kidney disease**					
Diabetic nephropathy	2 (40)	20 (25.3)	2 (28.4)	2 (12.5)	59 (35.3)
Hypertensive nephropathy	0	9 (11.4)	1 (14.3)	4 (25)	24 (14.4)
Glomerular disease	1 (20)	14 (17.7)	1 (14.3)	3 (18.8)	27 (16.2)
Polycystic kidney disease	0	2 (2.5)	1 (14.3)	0	6 (3.6)
Other	1 (20)	19 (24.1)	1 (14.3)	3 (18.8)	37 (22.2)
Unknown	1 (20)	15 (19)	1 (14.3)	4 (25)	14 (8.4)
Cardiovascular disease	3 (60)	46 (58.2)	5 (71.4)	8 (50)	101 (60.5)
Congestive heart failure	2 (40)	27 (34.2)	1 (14.3)	8 (50)	60 (35.9)
Diabetes mellitus	3 (60)	31 (39.2)	3 (42.9)	8 (50)	77 (46.1)
Arterial hypertension	5 (100)	70 (88.6)	7 (100)	13 (81.3)	157 (94)
Kidney transplantation	0	9 (11.4)	1 (14.3)	4 (25)	20 (12)
Immunosuppression	2 (40)	10 (12.7)	0	2 (12.5)	14 (8.4)
Pulmonary disease	2 (40)	21 (26.6)	3 (42.9)	2 (12.5)	30 (18)
Dialysis vintage (months)	3 (0.5–87)	31 (8–50)	4 (0–82)	27.5 (10.8–51.3)	34 (12–62)
Previous COVID-19	0	0	0	0	17 (10.2)
SARS-CoV-2 Sequencing	0	0	7 (100)	12 (75)	106 (63.5)
**Vaccination**					
1 dose	0	3 (3.8)	1 (14.3)	0	0
2 dose	0	0	2 (28.6)	7 (43.8)	19 (11.4)
3 dose	0	0	0	4 (25)	126 (75.5)
4 dose	0	0	0	0	4 (2.4)
Unvaccinated	5 (100)	76 (94.9)	3 (42.8)	4 (25)	15 (9)
Missing information	0	1 (1.3)	0	1 (6.3)	3 (1.8)
Duration PCR positivity (days)	10 (4–27)	12 (7–19)	20 (8–23)	12.5 (7.25–18.75)	7 (3–11)
Hospitalization	3 (60)	46 (58.2)	7 (100)	11 (68.8)	28 (16.9)
Antibiotics	5 (100)	61 (77.2)	7 (100)	13 (81.3)	56 (33.5)
Corticosteroids	0	26 (32.9)	6 (85.7)	8 (50)	13 (7.8)
Convalescent plasma	0	0	1 (14.3)	0	0
Remdesivir	0	2 (2.5)	2 (28.6)	3 (18.8)	3 (1.8)
Anti-SARS-CoV-2 Antibodies	0	0	0	0	3 (1.8)
Duration of hospitalization (days)	7 (6,7)	7.5 (4–19)	14 (5–28)	11 (7–25)	10 (6–17.75)
ICU admission	0	8 (10.1)	1 (14.3)	1 (6.3)	3 (1.8)
Mortality	1 (20)	17 (21.5)	3 (42.9)	4 (25)	2 (1.2)

^a^date of first positive PCR test to last PCR positive test within individual waves.

^b^BMI: One value missing in wave 2 and two values missing in wave 5.

The first wave included only five patients at our center, with one fatal case and three patients being hospitalized. The second wave started in October 2020 and ended in March 2021, and 79 SARS-CoV-2 positive HD patients were documented. Hospitalization, ICU admission and mortality were frequent events with 46 (58%), eight (10%) and 17 (21.5%) cases, respectively.

Rollout of vaccines in Austria commenced in early 2021. The first breakthrough infections were recorded during the third wave from 16th of March 2021 to 21st of May 2021. During this period, SARS-CoV-2 sequencing became available at our institution and revealed that the dominant variant infecting our patients was B.1.1.7 (Alpha). All patients infected with the Alpha variant had to be hospitalized. Mortality was particularly high during this wave (*N* = 3, 42.9%). During the following Delta wave, as apparent from sequencing results, 16 patients were infected. Hospitalization and mortality rates remained high at 68.8% and 25%, respectively. In contrast to previous waves, most patients (68.8%) had received two or more doses of vaccination at the time of infection.

Omicron became dominant in January 2022 and included the largest number of infected individuals with 167 PCR confirmed SARS-CoV-2 cases. A strikingly lower number of hospitalizations (*N* = 28, 16.9%) and mortality (*N* = 2, 1.2%) was seen during this wave. We also observed a decrease in the duration of PCR positivity. From a median of 20 days in the second wave, median positivity diminished to seven days in the fifth wave. Similarly, use of antibiotics in infected individuals was halved in the latter stages of the pandemic compared to earlier waves (77.2–100% compared to 33.5%). Corticosteroids, which are recommended in patients with oxygen dependency during infection and may therefore indicate severe disease, were more frequent in earlier waves compared to the Omicron era (32.9–85.7% vs. 7.8%). Other treatments, like antivirals, convalescent plasma or anti-SARS-CoV-2 antibodies were rarely used in our population. Notably, clinical characteristics and prevalence of pre-existing conditions were similar between waves.

### 3.2. Infection with the Omicron variant and vaccination are negative predictors of severe COVID-19

Next, we aimed to evaluate risk factors for hospitalization, ICU admission and mortality in all SARS-CoV-2 positive HD patients.

In univariate logistic regression, older age (OR 1.110, 95% CI: 1.011–1.219), diabetes (OR 1.882, 95% CI: 1.138–3.115), and pulmonary disease (OR 2.273, 95% CI: 1.257–4.102) were associated with an increased risk of hospitalization. Contrarily, vaccination (OR 0.580, 95% CI: 0.480–0.701) and infection during the Omicron era (compared to infections during previous waves) (OR 0.120, 95% CI: 0.068–0.211) conferred protection from hospitalization. Although SARS-CoV-2 reinfections were infrequent (*N* = 17), we observed a trend indicating a protective signal for reduced hospitalization rates (OR 0.235, 95% CI: 0.053–1.051) (Table 2). In a multivariable analysis diabetes and pulmonary disease were robust predictors for hospitalization in SARS-CoV-2 infected HD patients, whereas infection with an Omicron variant was associated with improved outcome and an almost 10-fold decrease of risk for hospitalization (Table 3). For ICU admission, which was overall a rare event in our cohort (*N* = 13), vaccination (OR 0.560, 95% CI: 0.353–0.888) and Omicron variant infection (OR 0.177, 95% CI: 0.048–0.661) were protective in univariate analysis (Table 2). However, they did not prevail in a multivariable analysis (Table 3).

**Table 2:** Univariate logistic regression for hospitalization.

	**Outcome variable**								
	**Hospitalization**			**ICU Admission**			**Mortality**		
Variable	Odds ratio	95% confidence interval	*p*-Value	Odds ratio	95% confidence interval	*p*-Value	Odds ratio	95% confidence interval	*p*-Value
Age (per 5 years increase)	1.110	1.011–1.219	0.029	0.963	0.798–1.161	0.689	1.23	1.029–1.471	0.023
Female Gender (compared to male)	1.021	0.620–1.681	0.936	1.392	0.455–4.255	0.562	2.152	0.947–4.889	0.067
BMI (per 1 kg/m^2^ increase)^a^	0.970	0.927–1.015	0.181	1.023	0.934–1.122	0.622	0.966	0.894–1.043	0.377
Kidney disease^b^	1.092	0.663–1.800	0.73	0.353	0.095–1.310	0.12	0.483	0.204–1.146	0.099
Cardiovascular disease	1.033	0.622–1.716	0.900	0.568	0.186–1.737	0.321	1.407	0.608–3.257	0.425
Heart failure	1.234	0.737–2.066	0.424	1.129	0.359–3.550	0.836	2.470	1.106–5.517	0.027
Diabetes	1.883	1.138–3.115	0.014	1.481	0.484–4.528	0.491	2.299	1.012–5.224	0.047
Arterial hypertension	1.456	0.550–3.853	0.449	1.050	0.130–8.474	0.963	0.325	0.109–0.966	0.043
Kidney transplantation	1.075	0.518–2.233	0.846	0.538	0.068–4.268	0.558	0.811	0.231–2.843	0.743
Pulmonary disease	2.273	1.257–4.102	0.006	2.453	0.771–7.804	0.129	2.926	1.275–6.715	0.011
Immunosuppression	0.731	0.309–1.729	0.476	0.722	0.090–5.772	0.759	0.680	0.152–3.037	0.613
Previous SARS-CoV-2 positivity	0.235	0.053–1.051	0.058		NA			NA	
Dialysis vintage (per 1 month increase)	0.995	0.990–1.001	0.096	0.991	0.975–1.008	0.319	1.003	0.997–1.008	0.335
Vaccination prior infection (as metric variable)^c^	0.580	0.480–0.701	< 0.001	0.560	0.353–0.888	0.014	0.468	0.325–0.674	< 0.001
Infection during Omicron Wave (compared to prior Waves)	0.120	0.068–0.211	< 0.001	0.177	0.048–0.661	0.01	0.040	0.009–0.172	< 0.001

ICU admission and mortality.

^a^ three cases missing.

^b^ diabetic/hypertensive nephropathy compared to other kidney disease.

^c^five cases missing.

NA, not applicable.

**Table 3:** Multivariable logistic regression for hospitalization.

**Multivariable logistic regression**			
Hospitalization			
	Odds ratio	95% confidence interval	*p*-Value
Age (per 5 years increase)	1.048	0.940–1.169	0.401
Diabetes	2.660	1.422–4.977	0.002
Pulmonary disease	2.329	1.171–4.632	0.016
Vaccination prior infection (as metric variable)^a^	0.994	0.684–1.443	0.973
Infection during Omicron Wave (compared to prior Waves)	0.108	0.037–0.316	< 0.001
ICU admission			
	Odds ratio	95% confidence interval	*p*-Value
Vaccination prior infection (as metric variable)^a^	0.757	0.369–1.551	0.447
Infection during Omicron Wave (compared to prior Waves)	0.331	0.044–2.504	0.284
Mortality			
	Odds ratio	95% Confidence interval	*p-*Value
Age (per 5 years increase)	1.159	0.937–1.434	0.173
Congestive heart failure	2.381	0.908–6.247	0.078
Diabetes	4.588	1.569–13.414	0.005
Hypertension	0.160	0.037–9.082	0.014
Pulmonary disease	3.250	1.163–9.082	0.025
Vaccination prior infection (as metric variable) ^a^	0.935	0.524–1.666	0.819
Infection during Omicron Wave (compared to prior Waves)	0.037	0.005–0.277	0.001

ICU admission and mortality.

^a^five cases missing.

Finally, age (OR 1.23, 95% CI 1.029–1.471), heart failure (OR 2.470, 95% CI: 1.106–5.517), diabetes (OR 2.299, 95% CI 1.012–5.224) and pulmonary disease (OR 2.926, 95% CI: 1.275–6.715) were positively associated with mortality in SARS-CoV-2 patients. Again, vaccination prior to infection (OR 0.468, 95% CI 0.325–0.674) and Omicron variant infection (OR 0.040, 95% CI 0.009–0.172) were significant negative predictors. Intriguingly, arterial hypertension lowered the risk for mortality (OR 0.325, 95% CI 0.109–0.966) (Table 2). Pulmonary disease, arterial hypertension, and Omicron variant infection remained significant in multivariable analysis, and the latter provides an approximately 20-fold risk reduction (Table 3). While vaccination appeared to provide protection from hospitalization, ICU admission and mortality in univariate analyses, when accounting for other variables, particularly Omicron variant infection, the previously observed beneficial effect of prior vaccination was no longer evident (Table 3).

### 3.3. At-risk hemodialysis population remained stable between 31st of August 2021 and 31st of August 2022 and displayed a high vaccination coverage

**Figure 3:**
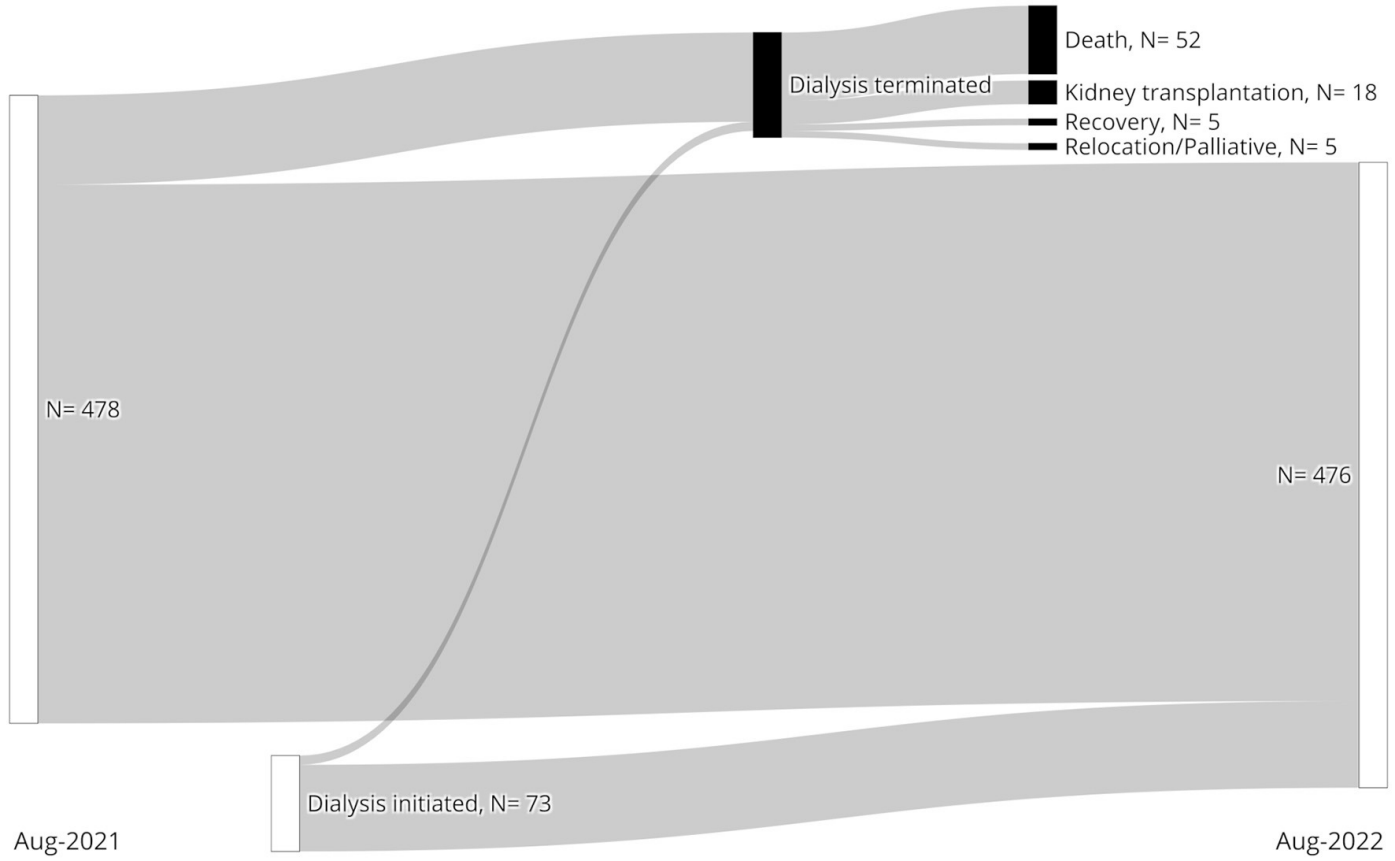
Dynamics of the at-risk hemodialysis population between 31st of August 2021 (**Left**) and 31st of August 2022 (**Right**) are shown.

To further evaluate the at-risk population and dynamics of vaccination coverage, we collected data from HD patients in Styria, Austria between 31st of August 2021 and 31st of August 2022 (Table 4). The included dialysis centers are shown in Figure 1. A total of 551 individuals met our inclusion criteria. Over 1 year, the HD population remained stable suggesting no excessive mortality although we lack comparative data from previous years (Figure 3).

**Table 4:** Clinical and vaccination data of hemodialysis patients on the 31st auf August 2021 and the 31st of August 2022 in Styria, Austria.

	**31-Aug-2021**	**31-Aug-2022**
N	478	476
		
Age (years)	70 (59–77.3)	71 (60–78)
Male gender (%)	291 (60.9)	296 (62.2)
BMI (kg/m^2^)^a^	25.9 (22.7–29.8)	26 (22.8–30)
Kidney disease		
Diabetic nephropathy	164 (34.3)	168 (35.3)
Hypertensive nephropathy	72 (15.1)	67 (14.1)
Glomerular disease	72 (15.1)	72 (15.1)
Polycystic kidney disease	28 (5.9)	30 (6.3)
Other	109 (22.8)	111 (23.3)
Unknown	33 (6.9)	28 (5.9)
Cardiovascular disease	319 (66.7)	306 (64.3)
Congestive heart failure	186 (38.9)	188 (39.5)
Diabetes mellitus	222 (46.4)	223 (46.8)
Arterial hypertension	448 (93.7)	448 (94.1)
Kidney transplantation	63 (13.2)	62 (13)
Immunosuppression	45 (9.4)	48 (10.1)
Pulmonary disease	96 (20.1)	87 (18.3)
Dialysis vintage (months)	30.5 (12–66)	35 (18–67)
Previous SARS-CoV-2 positivity	66 (13.8)	216 (45.4)
		
Vaccination^b^		
1 dose	11 (2.3)	1 (0.2)
2 dose	381 (79.7)	27 (5.7)
3 dose	3 (0.6)	225 (47.3)
4 dose	0	165 (34.7)
Unvaccinated	43 (9)	27 (5.7)
mRNA-1273/BNT162b2^c^	321 (81.3)/70 (17.7)	317 (75.8)/87 (20.8)

^a^Seven and eight cases missing in the 31-Aug-2021 and 31-Aug-2022 group, respectively.

^b^Missing information in 40 and 31 patients, respectively.

^c^Vaccine type referring to first dose. Percentages of vaccinated individuals at specified timepoint are given.

**Figure 4:**
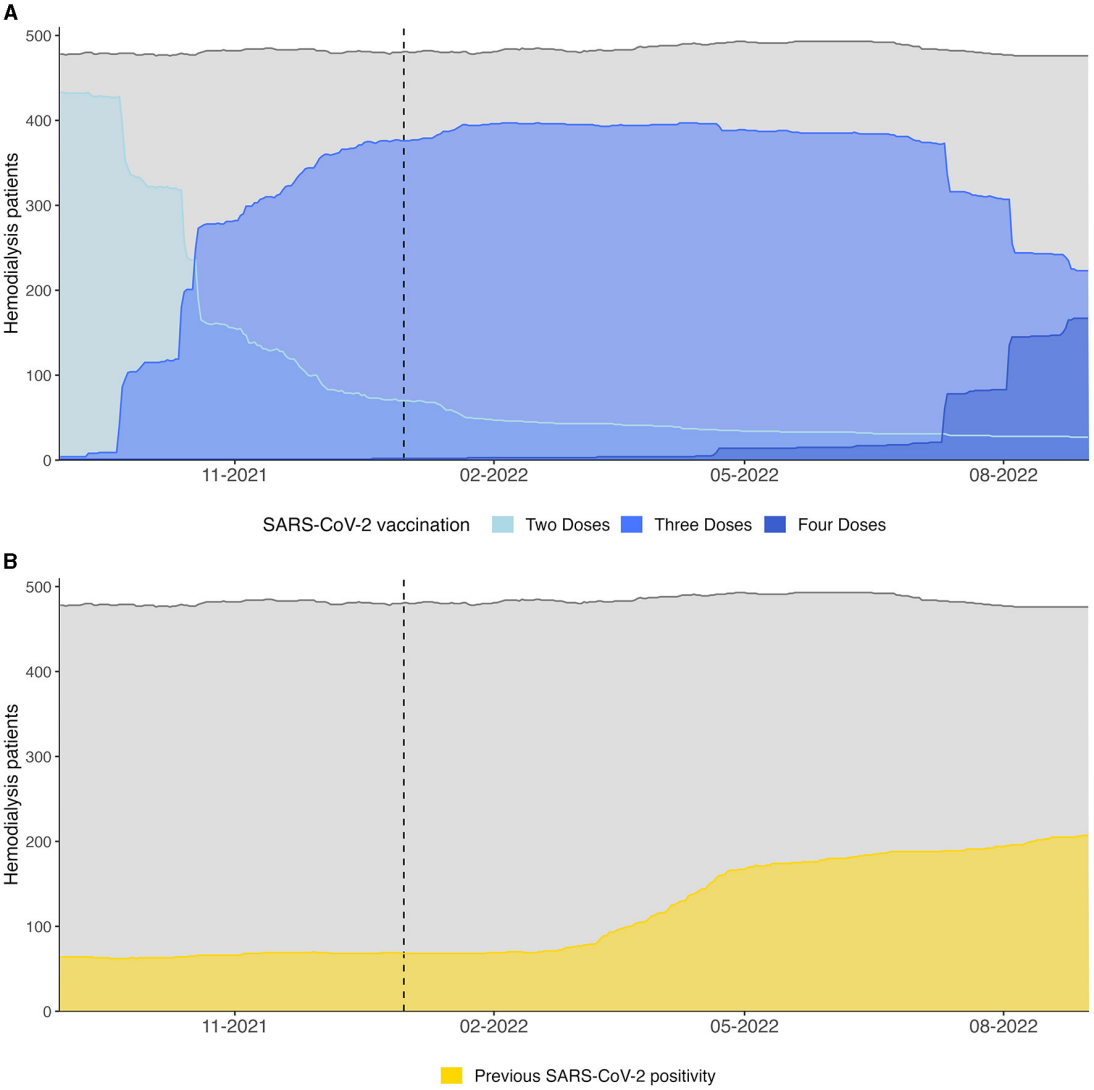
**(A)** Vaccination coverage and **(B)** proportion of recovered HD patients (defined as 28 days after the first PCR positivity) of the at-risk hemodialysis population between 31st of August 2021 and 31st of August 2022 are shown. Gray lines indicate the total number of dialysis patients. The dotted vertical lines indicate the switch from Delta to Omicron wave.

Vaccination coverage with at least two doses was around 80% in our population at the beginning of the observational period (Table 4, Figure 4A). By the end of 2021 the majority had received a third booster and by the end of August 2022 the number of four dose vaccinated dialysis patients were climbing (*N* = 165, 34%). Over 90% received mRNA-based vaccination.

At the same time a rapid increase in SARS-CoV-2 infections in our population was observed, which resulted in an approximately three times larger proportion of recovered individuals after 1 year compared to August 2021 (Table 4). Dynamics of the recovered patients, defined as 28 days after the first positive PCR test, a timepoint at which an antibody response following natural infection should be measurable (18), are shown in Figure 4B.

Our data suggests that Delta and Omicron waves challenged a rapidly changing population with regards to vaccination and infectious history, which resulted in a different vulnerability to severe COVID-19 (Figure 4).

### 3.4. Incidence and prevalence of SARS-CoV-2 positivity peaked with BA.1/BA.2

Next, weekly prevalence and incidence of SARS-CoV-2 infections were calculated by comparing the at-risk population to the SARS-CoV-2 positive and newly positive patients, respectively. When plotted against time, distinct waves become apparent. The separation of waves is further supported by sequencing data, which allows the differentiation of two distinct Omicron subwaves: the earlier BA.1/BA.2 subwave, which was replaced by end of May 2022 by the BA.4/5 subvariant. Omicron infections were preceded by the Delta wave until January 2022. Sequencing results are summarized in Table 4.

**Figure 5:**
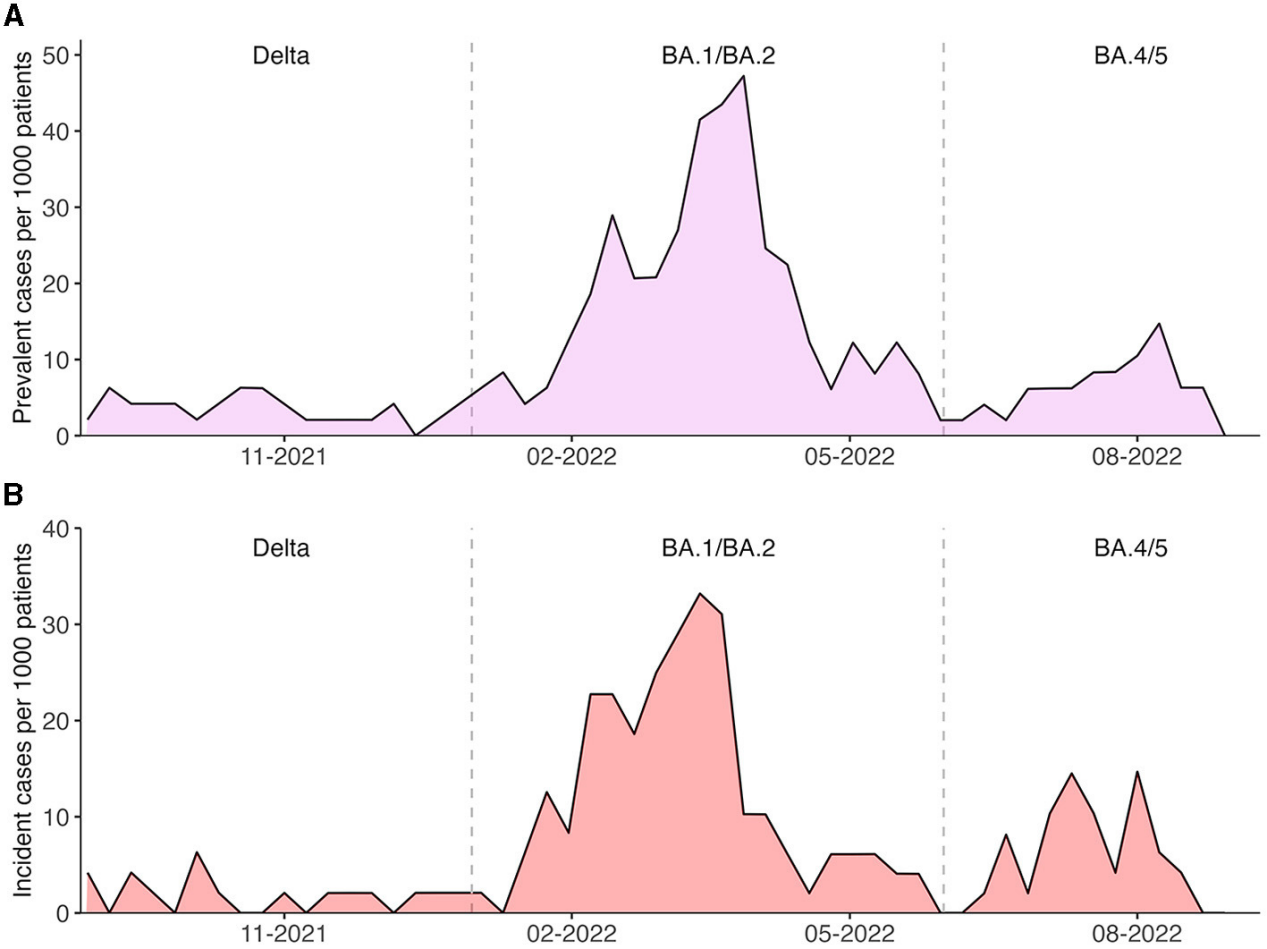
**(A)** Prevalence and **(B)** incidence of SARS-CoV-2 positive cases on hemodialysis between 31st of August 2021 and 31st of August 2022. The dotted lines mark the switch from Delta to BA.1/BA.2 and from BA.1/BA.2 to BA.4/5 infections, respectively.

Prevalence and incidence peaked during the BA.1/BA.2 dominated subwave with 47.2 and 33.2 per 1000 dialysis patients, respectively (Figures 5A, B).

Median prevalence during Delta, BA.1/BA.2 and BA.4/5 were 4.16, 12.4 and 6.21 per 1,000 dialysis patients, respectively (*p* ≤ 0.001). Median incidences were also significantly different between these three periods (Delta: 2.07 vs. BA.1/BA.2: 7.29 vs. BA.4/5: 4.20 per 1,000 dialysis patients, *p* ≤ 0.001).

### 3.5. BA.4/5 infections in hemodialysis patients remain equally mild as with BA.1/BA.2

Both Omicron subwaves differ substantially from previous waves, in that infected individuals were vaccinated more frequently (89.2% for BA.1/BA.2 and 89.2% for BA.4/5 with at least two vaccination doses at the time of infection). We also noted repeated COVID-19 in 6.2% and 24.3% for BA.1/BA.2 and BA.4/5, respectively, which underlines the profound immune escape displayed by the Omicron variant. Reinfections were mild with only two hospitalizations and no ICU admissions or deaths.

Severe disease necessitating hospitalization or ICU admission were rare events and similar in both Omicron subwaves (Table 5). However, duration of PCR positivity and hospital stay trended to be shorter in BA.4/5 compared to BA.1/BA.2. Mortality in both Omicron subwaves was almost non-existent and recorded only in one patient each (Table 5).

**Table 5:** SARS-CoV-2 PCR confirmed infections during the Omicron wave. Cases during the earlier BA.1/BA.2 dominated period are compared to the later BA.4/5 period.

	**BA.1/BA.2**	**BA.4/5**	
Time period	01-Jan-2022–31-May-2022	01-Jun-2022–31-Aug-2022	
N	130	37	*p*-Value
Age (years)	71 (55.8–78)	71 (58.5–77.5)	0.901
Male gender (%)	72 (55.4)	25 (67.6)	0.185
BMI (kg/m^2^)^a^	26.2 (23–30.8)	25.6 (22.2–29.8)	0.883
Kidney disease			0.729
Diabetic nephropathy	45 (34.6)	14 (37.8)	
Hypertensive nephropathy	17 (13.1)	7 (18.9)	
Glomerular disease	21 (16.2)	6 (16.2)	
Polycystic kidney disease	5 (3.8)	1 (2.7)	
Other	32 (24.6)	5 (13.5)	
Unknown	10 (7.7)	4 (10.8)	
Cardiovascular disease	76 (58.5)	25 (67.6)	0.318
Congestive heart failure	48 (36.9)	12 (32.4)	0.615
Diabetes mellitus	59 (45.4)	18 (48.6)	0.725
Arterial hypertension	121 (93.1)	36 (97.3)	0.340
Kidney transplantation	17 (13.1)	3 (8.1)	0.411
Immunosuppression	11 (8.5)	3 (8.1)	0.945
Pulmonary disease	22 (16.9)	8 (21.6)	0.511
Dialysis vintage (months)	34.5 (10.8–68)	32 (16.5–53.5)	0.967
Previous COVID-19	8 (6.2)	9 (24.3)	0.001
SARS-CoV-2 sequencing	85 (65.4)	21 (56.8)	
BA.1	36 (42.4)	0	< 0.001
BA.2	49 (57.6)	0	< 0.001
BA.4/5	0	21 (56.8)	< 0.001
Vaccination^b^			< 0.001
1 dose	0	0	
2 dose	18 (13.8)	1 (2.7)	
3 dose	98 (75.4)	28 (75.7)	
4 dose	0	4 (10.8)	
Unvaccinated	12 (9.2)	3 (8.1)	
Duration PCR positivity (days)	7 (3-12)	5 (3–8)	0.130
Hospitalization	21 (16.2)	7 (18.9)	0.691
Antibiotics	45 (34.6)	11 (29.7)	0.579
Corticosteroids	11 (8.5)	2 (5.4)	0.540
Remdesivir	2 (1.5)	1 (2.7)	0.638
Anti-SARS-CoV-2 Antibodies	3 (2.3)	0	0.351
Duration of hospitalization (days)	13 (8–19)	5 (3–9)	0.077
ICU admission	3 (2.3)	0	0.351
Mortality	1 (0.8)	1 (2.7)	0.340

^a^one value missing in each group.

^b^missing information in 2 and 1 cases, respectively.

## 4. Discussion

COVID-19 has posed a great threat to the lives of HD patients in the early pandemic (2–4), who were often particularly exposed due to regular in-center HD (1). Compared to the general population, diminished antibody response to SARS-CoV-2 has been shown in HD patients (14), leaving them vulnerable after infection and vaccination (12, 19).

Presently, we analyzed COVID-19 cases at our center from the first recorded case in March 2020 until August 2022. In agreement with existing reports, we could show the high morbidity and mortality associated with COVID-19 in the early stages of the pandemic. Furthermore, we could confirm that the Omicron variant has been highly prevalent in dialysis patients, but virulence has been markedly lower than in previous waves. Our at-risk population was extensively vaccinated and exhibited a strong willingness for a third and fourth dose. We also report that infections with Omicron sublineage BA.4/5 do not differ from Omicron sublineages BA.1 and BA.2 with regards to hospitalization, ICU admission, duration of PCR positivity and mortality. We think that our study provides valuable information for nephrologists, who are concerned with these novel sublineages.

High prevalence of comorbidities may render HD patients susceptible to severe and fatal COVID-19. Important risk factors include older age, diabetes, hypertension, and cardiovascular disease (20). In agreement, older age, heart failure and pulmonary disease were predictors of COVID-19 related mortality in our population. However, Ng et al. have shown, that even after adjustment for concomitant disease, end-stage kidney disease remains a robust predictor of mortality (3). It is speculated, that uremic alterations of the innate and adaptive immune response may predispose to infections (21).

With reduced virulence in latter stages of the pandemic, and with the emergence of Omicron as the dominant variant, mortality has become a rare event (8). Congruently, hospitalization rates declined significantly (22). Nevertheless, due to the high transmissibility of Omicron (9), the total number of hospitalized COVID-19 cases remained high. Even though hospitalization in the Omicron era was necessary in only 16.9% of cases, the absolute number exceeded all previous waves except for the second wave. Although the need for hospitalization is subject of the clinician's assessment and therefore not a strictly objective outcome, it is highly relevant as it poses a substantial cost factor for health care systems. Duration of hospitalization remained similar between waves, and prolonged viral shedding has been described in patients with impaired kidney function further adding to the problem (23). Thus, despite largely losing the threat of a life-threatening disease, COVID-19 still has the potential to overwhelm health care providers.

We report a low number of ICU admissions, which may reflect triage as these patients were often deemed to have no recovery potential. Therefore, caution is warranted when interpreting ICU admission as an outcome parameter of severe disease in the HD population. A recent review found inconsistent evidence regarding ICU admissions in CKD patients, while hospitalization and mortality were robustly increased in CKD with COVID-19 compared to non-CKD (24). Although it is difficult to estimate the “true” need for intensive care, Chan et al. reported that the rate of ICU admissions in HD patients was only about 9% compared to 21% in a propensity score matched control group, despite comparable burden of comorbidities and similar symptoms at hospital admission (25).

In agreement with others, we clearly show that the threat for HD patients has progressively diminished over the course of the pandemic (7). Since the emergence of SARS-CoV-2, several important changes ought to be highlighted: first, vaccinations are a safe and effective measure in the prevention of severe disease. Second, treatments have been developed to reduce mortality in those already infected. Third, accumulating infections resulted in a certain degree of natural immunity among survivors, potentially mitigating viral transmission and/or disease severity. Finally, VoCs have profoundly altered the pandemic in terms of transmission dynamics and disease severity. These changes largely coincided with each other, thus making it challenging to quantify the contribution of each individual factor.

Even prior to the emergence of these factors, COVID-19 related morbidity and mortality decreased in the HD population (26), which may simply be a consequence of more widespread testing and the identification of more oligo- and asymptomatic patients (26). Whether the at-risk population was altered with the particularly vulnerable already having succumbed to the initial wave of SARS-CoV-2 remains debated (27, 28). Our at-risk HD population between August 2021 and August 2022 remained stable and prevalence of comorbidities was comparable at both timepoints.

Immune-escape is another hallmark of Omicron (10). While repeated antigenic stimulation by booster vaccination appears to provide some protection from infection (15), neutralizing activity against Omicron BA.1 remains insufficient even after four doses (29). Despite the high vaccination coverage in our dialysis population, we saw a massive surge in infections in 2022. Previous vaccination conferred protection from hospitalization and mortality in our study only in univariate analysis. When controlled for other factors, especially timing of infection (pre-Omicron vs. Omicron era) the protective effect of vaccination disappeared. These findings may be attributable to the profound immune escape displayed by Omicron sublineages. The novel bivalent Omicron BA.4/5-adapated vaccine elicits a robust response in HD patients and may offer improved protection from these sublineages (30). Of note, these adapted vaccines were rolled-out after the end of our observational period in Austria.

Before the advent of vaccines, natural infection was the only way to acquire anti-SARS-CoV-2 antibodies, which have been shown to protect from reinfection in HD patients in the pre-Omicron era (12). A recent meta-analysis concluded that the risk of reinfection in the general population with Omicron sublineages is substantially higher than with previous variants, but natural infection still offers a certain degree of protection especially from severe disease (31). We observed 17 mild reinfections in our cohort, and previous SARS-CoV-2 infection tended to be a protective factor against hospitalization (Table 2). Analysis of the impact on ICU admission and mortality was hindered by the low number of reinfections and events. Reinfections were noted exclusively during the Omicron wave. Since we did not assess for antibody titers, we can only speculate that those individuals either failed to mount a substantial humoral response during the earlier infection or were affected by Omicron's heightened immune escape capabilities. Existing data suggests, that the level of protection against BA.4/5 is approximately twice as high when BA.1 was the previous infection compared to pre-Omicron variants (31). Thus, it is tempting to speculate that the increasing number of recovered HD patients during Omicron may have limited further viral spread (Figure 4B). Importantly, the protective efficacy of natural infection compared to vaccination in terms of protection from subsequent Omicron and Omicron sublineage infection in HD patients remains uncertain.

A major strength of this study is the comprehensive and well characterized cohort of HD patients, which was followed over the course of 1 year. Large registry studies have previously reported on COVID-19 in HD patients, but either during a limited observational period (7, 32), or before the emergence of Omicron as dominant variant (33–35). Our study depicts a rapidly changing at-risk population by including extensive information on natural and induced immunity by previous infection and vaccination, respectively. This provides a more complete picture of the real-world impact of the pandemic on the vulnerable HD population. We were also able to characterize and compare infections with BA.1/BA.2 and BA.4/5 in a sizeable number of HD patients.

Apart from the retrospective nature of our study, which comes with inherent bias, our study is limited by its comparatively small population, which may have limited our ability to detect differences especially when comparing smaller waves. While we separated distinct waves based on time and sequencing data, sequencing was not available in all patients. Despite rigorous antigen testing of asymptomatic individuals, we cannot exclude the possibility of undiagnosed SARS-CoV-2 infections, particularly in earlier waves (36). We also acknowledge the reduced sensitivity of antigen testing compared to PCR especially in asymptomatic individuals (37). Yet, diagnostic yield may have been greater in our population due to twice or thrice weekly testing before each dialysis session. We only counted infections if there was evidence within the electronical health records of PCR confirmed SARS-CoV-2 positivity. However, this may have underestimated the number of previous infections in those who became dialysis-dependent later during the pandemic, as PCR results may not have been available, or they may have not been tested as frequently.

While optimization of vaccines and treatments is ongoing, viruses, as well, undergo constant mutations, which may result in the emergence of novel variants and sublineages. Rapid information on new variants or sublineages is paramount to prepare for effective prevention and treatment especially for the vulnerable HD population.

Our findings underline the reduced virulence but increased transmissibility of Omicron in HD patients. Furthermore, we showed that infections with Omicron sublineages BA.4/5 are similarly mild as with BA.1 and BA.2 in HD patients. Although our data is reassuring to clinicians that the situation will remain calm with BA.4/5, we simultaneously acknowledge the importance to remain vigilant for the emergence and spread of novel variants.

## Data availability statement

The original contributions presented in the study are included in the article/supplementary material, further inquiries can be directed to the corresponding author.

## Ethics statement

The studies involving humans were approved by the study was approved by the Ethics Committee of the Medical University of Graz (EK 34-372ex21/22). The studies were conducted in accordance with the local legislation and institutional requirements. Written informed consent for participation was not required from the participants or the participants' legal guardians/next of kin because retrospective analysis of dialysis cohort. No potentially identifiable images or data are presented in this study.

## Author contributions

MS designed the study and interpreted and analyzed the data, drafted the work, finally approved the manuscript, and agreed to be accountable for all aspects of the work. NG, AP, MW, MK, NS, CS, KM, and HH-G acquired the data, revised the manuscript, finally approved the manuscript, and agreed to be accountable for all aspects of the work. AR and PE interpreted the data, revised the manuscript, finally approved the manuscript, and agreed to be accountable for all aspects of the work. KE designed the study and interpreted the data, revised the manuscript, finally approved the manuscript, and agreed to be accountable for all aspects of the work. All authors contributed to the article and approved the submitted version.
